# On the Importance of Relative Salience: Comparing Overt Selection Behavior of Single versus Simultaneously Presented Stimuli

**DOI:** 10.1371/journal.pone.0099707

**Published:** 2014-06-13

**Authors:** Alisha Siebold, Mieke Donk

**Affiliations:** Department of Cognitive Psychology, Free University, Amsterdam, the Netherlands; University of Groningen, Netherlands

## Abstract

The goal of the current study was to investigate time-dependent effects of the number of targets presented and its interaction with stimulus salience on oculomotor selection performance. To this end, observers were asked to make a speeded eye movement to a target orientation singleton embedded in a homogeneous background of vertically oriented lines. In Experiment 1, either one or two physically identical targets were presented, whereas in Experiment 2 an additional orientation-based salience manipulation was performed. The results showed that the probability of a singleton being available for selection is reduced in the presence of an identical singleton (Experiment 1) and that this effect is modulated by the salience of the other singleton (Experiment 2). While the absolute orientation contrast of a target relative to the background contributed to the probability that it is available for selection, the crucial factor affecting selection was the relative salience between singletons. These findings are incompatible with a processing speed account, which highlights the importance of visibility and claims that a certain singleton identity has a unique speed with which it can be processed. In contrast, the finding that the number of targets presented affected a target's availability suggests an important role of the broader display context in determining oculomotor selection performance.

## Introduction

Over the last couple of years studies on visual attention and eye movement behavior aimed to explain selection behavior not traditionally in terms of either purely stimulus-driven [1]–[3] or goal-driven processes [4]–[9] but rather in terms of how the two processes interact in determining selection preferences [10]–[16]. Most importantly, the relative contribution of each process was found to vary as a function of the timing of a response relative to the onset of a stimulus display. For instance, Donk and van Zoest (2008) varied salience by presenting observers with two differently oriented line segments relative to a background of homogeneously aligned lines and instructed them to make a single speeded eye movement to the most salient line in the display [17]. While the proportion of correct responses was initially high for eye movements elicited up to 250 ms following display onset, it dropped to chance level for slower responses. They concluded that overt selection is only salience driven for a brief period after stimulus presentation but does not guide selection beyond this crucial time window. Using a similar stimulus display in which the target was either more or less salient than the distractor, we recently corroborated these findings and additionally showed that 1) salience-driven processes initially dominate selection even when salience-based information is detrimental to the task and 2) that slower eye movements as well as subsequent eye movements are guided by goal-driven processes in line with task requirements, irrespective of target salience [18] (for complementary findings see also [17], [19]–[28]). These findings suggest that while stimulus-driven processes attract the eyes in an automatic fashion, time is required in order for goal-driven processes to set in, possibly as a result of feedback loops or recurrent processes interacting with hierarchically lower structures required to extract relevant information necessary for target identification [12]; [29]–[32].

An altogether different approach in explaining the patterns of results typically observed as a result of salience manipulations was proposed by De Vries, Hooge, Wiering, & Verstraten (2011) [33]. Rather than assuming that a salient element is prioritized over a less salient element at short response latencies, they proposed that differences in the absolute processing speed of individual elements underlie selection preferences. They reported a set of experiments, in which observers were presented with two superimposed grids, one sampled at a high- and one at a low-spatial frequency, containing an orientation target each, amongst a homogeneous background of vertically oriented lines. Observers were asked to make a speeded eye movement to any of the two orientation singletons. In line with previous findings [34]–[35] they observed that a greater number of responses was made towards the low-spatial frequency singleton for fast responses, arguing that it had been available and processed at an earlier point in time than the high-spatial frequency singleton. This preference disappeared for slower responses, resulting in an equal selection frequency for both singletons. They took these results as evidence that the processing time of an individual element is key in determining whether this element is selected. According to this approach, an element receives prioritized selection at an early point in time because it is the only element available to the visual system and therefore the only element being processed at this particular time. As time passes, multiple elements are processed, resulting in competition and the chances of any one element being selected are evenly distributed among the processed elements, explaining chance level performance for longer latency responses.

This theory fits nicely with the patterns of results reported above in which the selection probability of a salient target in the presence of a non-salient distractor decreases as saccadic latency increases. Nevertheless, de Vries et al. crucially differ with regard to the assumptions of the underlying mechanisms causing the drop in performance of selecting a salient element with increasing response latencies. A processing speed account [33] assumes that the drop in performance at around 250 ms post stimulus presentation is due to an increase in competition for selection between the two singletons, presumably because at this time both stimuli are available for processing to the visual system. On the other hand, a relative-salience account [17] attributes the decrease in performance of selecting a more salient singleton to the visual system being increasingly less sensitive to differences in relative salience at longer latencies. While a processing-speed approach is very intuitive (a singleton needs to be visible and therefore processed in order to be selected) and appealing given its parsimonious theoretical rationale, we recently showed that caution needs to be exercised in generalizing these findings to ones obtained from a context manipulation based on relative salience between elements. We presented observers with stimulus displays similar to de Vries et al. [36]. Crucially, one singleton, the fixed singleton, had an orientation contrast relative to the background lines that was identical across conditions whereas the other singleton was either more salient (it had a larger orientation contrast relative to the background lines) or less salient (it had a smaller orientation contrast relative to the background lines) than the fixed singleton. According to de Vries et al., the fixed singleton should have been available for oculomotor selection at the same point in time in both conditions since it had the same orientation contrast relative to the background lines. A relative salience account, on the other hand, assumes that the availability of the fixed singleton should vary contingent on the orientation contrast (salience) of the other singleton. Based on these diverging assumptions regarding the underlying mechanisms of overt visual selection behavior, we developed two alternative models corresponding to the two different accounts and showed that, while local feature contrast is clearly important, a model based on the relative salience between elements was better able to account for selection behavior than a model based on the processing speed between these elements alone. That is, the estimated availability of an individual singleton for selection was not merely a function of its own specific visibility, as determined by its feature contrast relative to its direct surroundings, but varied in dependency of the salience of the other simultaneously presented singleton. Accordingly, these results are a reminder of the importance of the concept of a salience map or a topographic representation of salience in the brain [37]–[39]. While the local feature contrast of a singleton, thus its visibility, contributed to selection performance, the relative salience between *all* items in a display rather than the local distinctiveness of each individual item was found to be the crucial factor in determining its availability for oculomotor selection and thus its individual selection probability. However, to date it is unknown how exactly the availability of an individual item to the oculomotor system is affected by the presence of another distant item, especially when salience between these items is manipulated. In case a single singleton is presented, the availability of this singleton is purely determined by differences in local feature contrast. By contrasting performance in response to single presentation of a singleton with performance in response to the same singleton in the presence of an additional singleton (either identical or different with respect to the salience relative to this singleton) it allows for a detailed investigation of the contribution of both local feature contrast and relative salience between elements to overt selection behavior.

Thus, the present study aimed to investigate how the availability of an individual singleton for oculomotor selection is affected by the presence of another distant singleton that is either equally salient (Experiment 1) or in which salience is varied between elements (Experiment 2). To this end, we presented observers with either one (single condition) or two simultaneously presented orientation singleton(s) (dual condition) amongst a homogeneous background of vertically aligned line segments. In Experiment 1 the local orientation contrast of the singletons relative to the background was identical (equally salient) whereas in Experiment 2 we added a stimulus salience manipulation by varying the local orientation contrast of the singleton(s) relative to the background (single and dual salient and non-salient conditions, respectively) and relative to each other (dual different condition). Observers had to make a speeded eye movement to the singleton in the single condition and to any of the two singletons in the dual conditions. We measured selection performance in terms of the selection frequencies of a specific orientation singleton in relation to its corresponding saccadic latencies. In both experiments, we separately calculated how a singleton's availability for selection varied in dependency of the presence of another distant singleton and how this availability was affected by the local feature contrast of the singletons.

## Experiment 1

The goal of Experiment 1 was to examine whether a singleton's availability for selection is differentially affected for single (single condition) compared to simultaneous presentation (dual condition). More specifically, we were interested in whether and how the availability of one particular orientation singleton is affected by the presence of an identical singleton.

A singleton's availability cannot be directly observed but can be inferred from the observed response frequencies, expressed as its underlying selection probability corrected for the number of alternative response options.

Given that both singletons in the dual condition were equally salient, we assumed that both had an identical availability and were, therefore, equally likely to be selected. According to a processing speed account, a singleton's availability for selection should be unaffected by the presence of another equally salient singleton. That is, there should be no difference in terms of availability between the singleton in the single and that in the dual condition. Alternatively, a singleton's availability might not only depend on its local feature contrast but might also depend on the presence of another singleton. In this case - even though the two singletons are equally salient - the mere presence of one singleton might lower the availability of the other singleton just because the presence of another equally salient singleton lowers the overall perceived salience of either of the two singletons compared to the presentation of that same singleton on its own. In that case, the availability of the singleton in the dual condition should be reduced compared to the availability in the single condition, as postulated by a relative salience account.

### Method

#### Ethics Statement

The present study, including the consent procedure, was approved by the Vaste Commissie Wetenschap en Ethiek (VCWE), the ethics board of the Faculty of Psychology and Education, and conducted according to the principles of the Declaration of Helsinki. Participants received information about the study and their rights and gave written informed consent.

#### Participants

Ten participants (including the author AS) took part in the experiment in return for either money or course credit. Ages ranged from 19 to 31 years (mean: 25.3 years); six of the participants were female. All participants reported normal or corrected-to-normal visual acuity and all apart from AS were naïve to the purpose of the study. The experimental session lasted for approximately 30 minutes.

#### Stimuli and Apparatus

**Figure 1:**
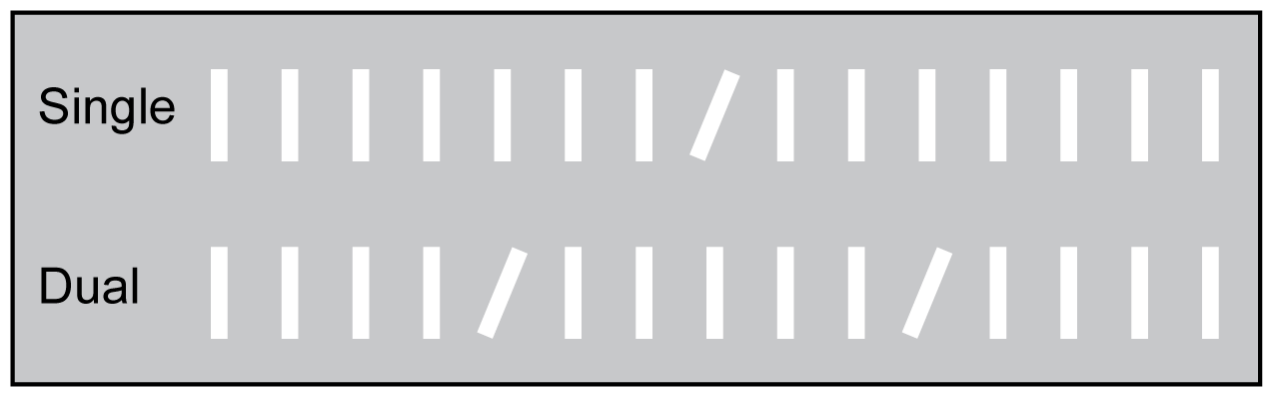
Stimulus overview of Experiment 1. The figure depicts a simplified overview of the different conditions in Experiment 1. In the single condition only one singleton is presented whereas in the dual condition two singletons are presented simultaneously. The singletons have an identical orientation contrast of 22.5° relative to the background lines across both conditions.

The visual stimuli consisted of 289 white lines (89.4 cd/m^2^) with a size of 0.76 * 0.15 cm each (each line covering an area of 1.4 * 0.2° of visual angle as measured at central fixation), contained in a 17 * 17 items square matrix, extending a total of 25.7° * 25.7° of visual angle. The displays featured either one or two singletons with a fixed orientation contrast of 22.5° clockwise relative to multiple homogeneously aligned background lines (90° relative to the horizontal plane), presented on a gray background screen (22.1 cd/m^2^). In the single condition, one orientation singleton was randomly presented at one of eight potential grid locations on an imaginary circle around the center of the grid at a distance of approximately 6°. In the dual condition, two identical orientation singletons (both tilted 22.5° clockwise relative to the background lines) were randomly presented at any of the eight grid locations with the constraint that the singletons were never positioned in adjacent locations in order to minimize the occurrence of a global effect and to be able to clearly classify eye movements as being directed toward either one of the two singletons. The minimal distance between the two singletons was approximately 5.7°. Figure 1 presents an overview of the different presentation conditions.

A standard Pentium IV class computer with a processor speed of 2.3 GHz running E-Prime 2.0 software package controlled stimulus presentation, timing of events, and acquisition of necessary response data. Stimuli were presented at eye level, 75 cm from the chinrest, on a 21-inch Iiyama SVGA (Super Video Graphics Array) monitor, running at 1024 * 768 pixel resolution with a 100 Hz refresh rate. The position of the right eye was recorded every 1 ms by means of an Eyelink 1000 eye tracker (SR Research Ltd., Mississauga, Ontario, Canada), with a 1000-Hz temporal resolution, a 0.01° of visual angle spatial resolution (noise limited), and a gaze position accuracy of 0.5°.

#### Design and Procedure

**Figure 2:**
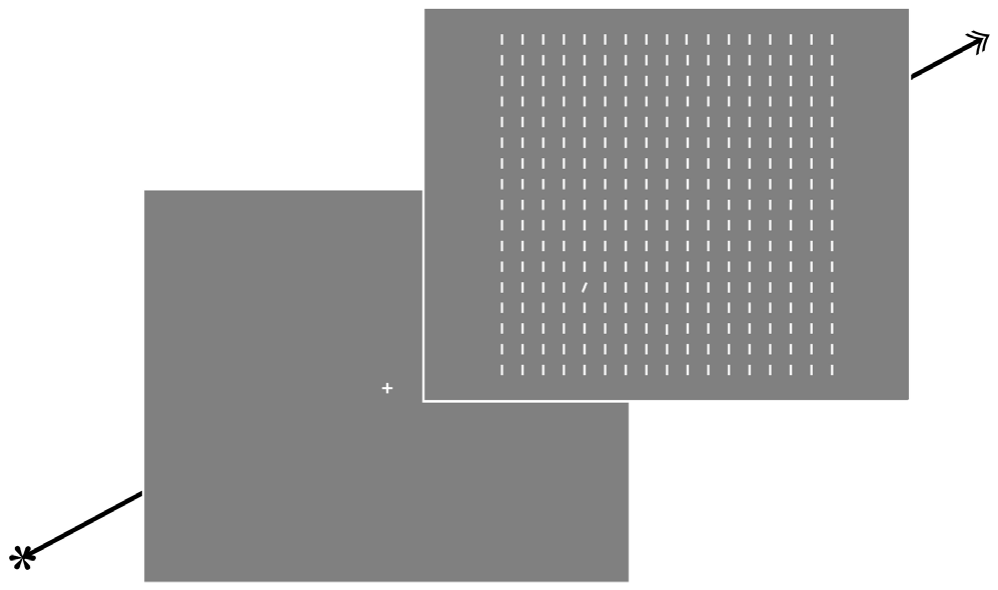
A typical trial sequence. Participants maintained fixation on a centrally presented cross until a stable fixation was detected. After pressing spacebar, a drift correction was performed and subsequently a trial was initiated. The search display was presented for 2000

Participants received instructions and information regarding the study before giving written consent. They were tested in a dimly lit sound-attenuated research lab. Prior to the testing session, eye movements were calibrated to a precision of 0.5° of visual angle. In the single condition participants were instructed to make a speeded eye movement to the only orientation singleton whereas in the dual presentation condition they were free to make a speeded eye movement to any of the two orientation singletons. Prior to the presentation of the search display, participants had to maintain fixation on a centrally presented white fixation cross and initiate a trial preceded by an automatic drift correction by pressing spacebar. The search display was presented for 2000 ms after which the screen was blanked and a fixation cross was presented to indicate the beginning of the next trial (for a depiction of a typical trial sequence see Figure 2). Participants performed 40 practice trials, which were not included in any analyses. They completed a total of 400 experimental trials, presented in random order across conditions and separated by four small breaks.

#### Data Analysis

Eye movements were filtered online and required a minimum peak velocity of 35°/s and a minimum acceleration of 9500°/s^2^ in order to be detected as a valid saccade. Subsequently, fixation coordinates and saccade timings and amplitudes of the initial saccades following display onset were extracted from the raw eye tracking data and were subjected to a MATLAB-based analysis protocol. Similarly to previous studies [18]; [36]; [40], a trial was discarded if the saccade amplitude remained below 2.3°, if a saccade did not land within the range of 3° of visual angle of any of the target singletons (denoted as saccade destination error) or if a blink occurred during the crucial time interval between display onset and selection of a singleton. Furthermore, in order to prevent anticipation and attention-lapse errors from biasing the results, trials in which the latency of the initial saccade was shorter than 80 ms or longer than 600 ms were likewise discarded. A participant's complete dataset was removed from analyses if more than 30% of all trials had to be discarded. The remaining saccades were classified as directed toward the singleton (in the single condition) or either of the two singletons (in the dual condition) and the total distribution of initial saccade latencies was rank-ordered from fastest to slowest responses across conditions but separately for each participant. The individual latency distributions were divided into five bins consisting of 20% percentiles each. Note, that while the division of trials into five separate bins is arbitrary, it is inspired by previous studies [18]; [22]; [33]; [36]; [40] having shown that this particular division adequately captures selection behavior as it evolves over time.

### Results

A total of 12% of all trials was excluded from analyses (0.9% due to anticipation errors, 0.3% due to attention-lapse errors, 7.3% due to saccade destination errors, and on 3.5% the amplitude remained below 2.3°). No erroneous blinks were recorded in the time interval from display onset until fixation of a singleton and none of the participant's datasets exceeded the critical removal criterion of 30%.

In order to examine differences in the timing of selection between the different presentation conditions, we performed a t-test comparing the saccadic latencies in the single to those in the dual condition. The results indicated no significant differences in timing of selection between the two conditions [*t*(9) = −.013, *p* = .990], suggesting that the speed with which participants selected a singleton did not differ between the single and dual condition.

**Figure 3:**
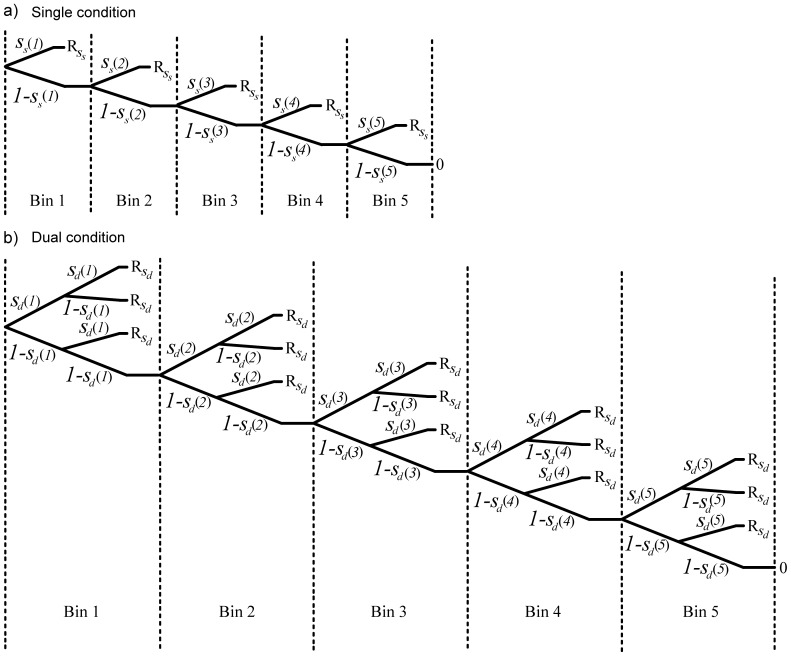
Tree diagrams in Experiment 1. The tree diagrams correspond to a) the single and b) the dual condition and depict the probabilities that a singleton is available for selection (italics) for each bin (in brackets), leading to a given response outcome (R) for each individual trial. The response outcome is determined through the path leading to it, which equals the product of probabilities on that path. 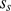 denotes the probability that the singleton is available in the single condition and 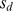 denotes the probability that the singleton is available in the dual condition.

In order to examine how the availability of an individual singleton for oculomotor selection varies when a singleton is presented in isolation compared to when it is simultaneously presented with another (identical) singleton, we calculated the availability of the singleton separately for the single and dual condition on the basis of the tree diagram depicted in Figure 3. Since the availability of a singleton to the visual system cannot be directly observed, it can be extrapolated based on the observed frequencies of responses to each singleton. For each participant and condition we first determined the probability that the singleton was available for selection for each bin (*i*) of the response time distribution (with 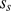 denoting the probability that the singleton is available for selection in the single condition, and 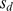 corresponding to the probability that the singleton is available in the dual condition). Note that the availability of both singletons is assumed to be identical. Therefore, the response frequencies of both singletons have been combined into 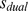.

It was assumed that the selection of a singleton in a given trial depends on the availability of the individual singleton for oculomotor selection at time (*i*) of selection. Depending on the availability of the singleton, different selection outcomes are possible. Since there is only one singleton present in the single condition, this singleton is either available and therefore selected at time (*i*), or selection is postponed to a later point in time given that it is not (yet) available [1 – 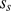]. In the dual condition, the potential outcomes are: a) both singletons 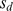 are available at the same time (*i*) and therefore equally likely to be selected [with probability 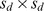], b) only one of the singletons is available while the other one is not [with probability 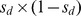], or c) none of the two singletons are available (yet) and selection is postponed to a later bin (*i*) [with probability 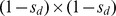]. The probability of a particular outcome in a given trial is determined by the sum of all branches leading to that outcome. The overall probability that a singleton is available in a given trial is separately calculated for each singleton and latency bin (1–5) based on the individual observed selection frequencies for each possible selection outcome. Note, that the parameter estimates derived from the model represent the probabilities that a given singleton is available for selection at a given point in time. These estimates represent the best fit of the model to the distribution of observed responses.

**Figure 4:**
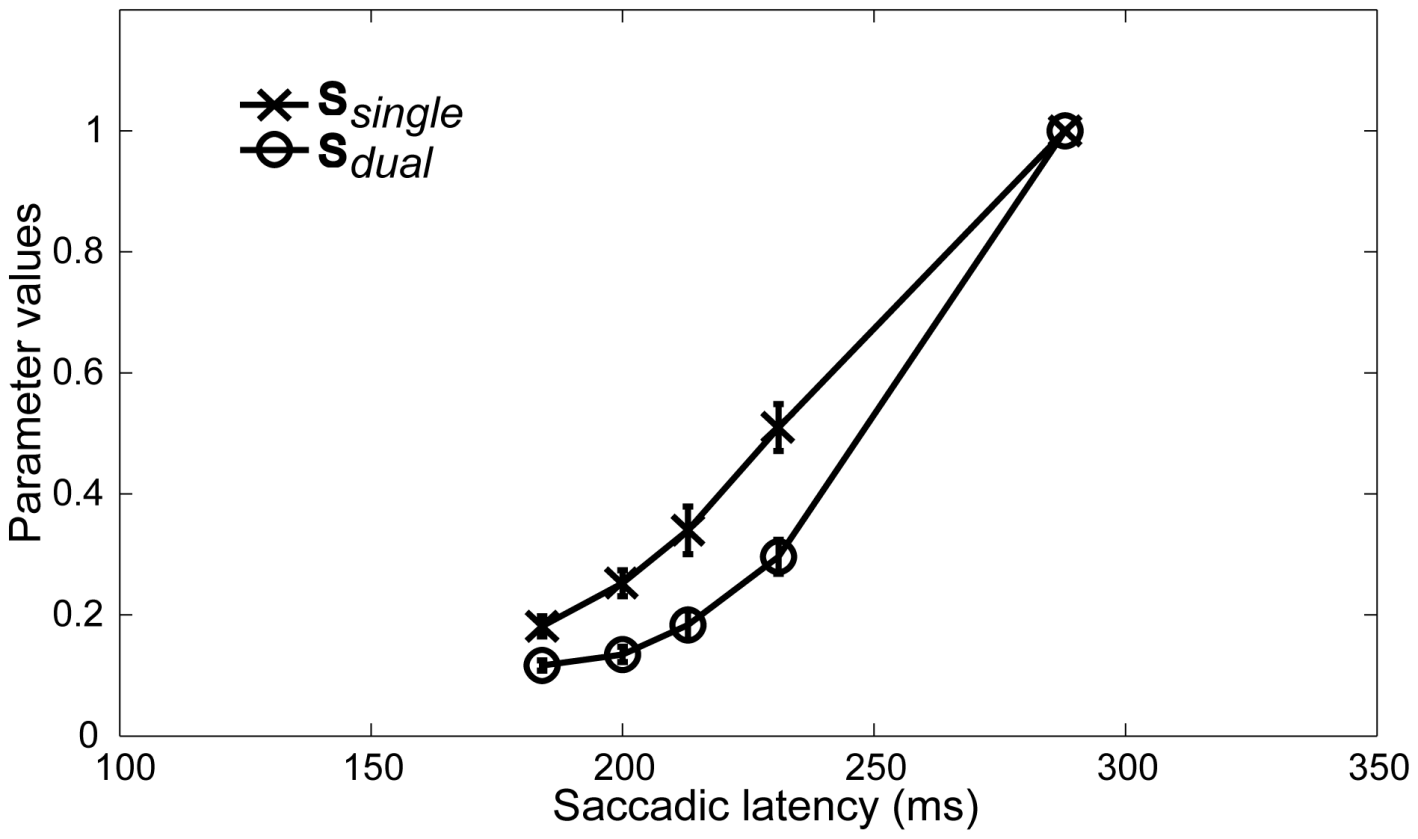
Parameter values in Experiment 1. The parameter values represent the probability that a singleton is available for selection separately per condition for each Bin *i*, averaged over participants. Error bars represent 95% confidence intervals. Note that based on the assumption that the availability of both singletons in the dual condition is identical, Bin 5 in this condition adds up to 1by definition.

In order to compare the probability that a singleton is available in the single to the probability that the same singleton is available in the dual condition, we performed a 2 (Condition) * 5 (Saccadic Latency Bin) ANOVA on the individual parameter values corresponding to 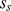 and 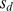. The results revealed a main effect of Condition [*F*(1, 9) = 41.890, *η* ^2^ = .030, *p*<.001] and Saccadic Latency Bin [*F*(4, 36) = 28133.926, *η* ^2^ = .945, *p*<.001] as well as a significant interaction between the two factors [*F*(4, 36) = 22.498, *η* ^2^ = .013, *p*<.001]. Post-hoc Bonferroni corrected comparisons revealed that overall the parameter values corresponding to the singleton in the single condition were larger [*m* = .46; *std error* = .010] than the values obtained for the same singleton in the dual condition [*m* = .35; *std error* = .007]. A graph of the results is shown in Figure 4.

### Discussion

The results of Experiment 1 showed that the availability of the singleton in the single condition was higher than in the dual condition. That is, the addition of an identical singleton in the dual condition led to a decrement in availability for selection relative to the single condition. This finding presents corroborating evidence against a processing speed account [33] according to which the probability that the singleton is available in the single condition should not differ from the probability that the singleton is available in the dual condition since all singletons had an identical orientation contrast relative to the background. Our findings suggest that the availability of an individual element is not a sole function of its local feature contrast, but is rather determined by the broader context, in this case, the presence of another equally salient singleton.

While the current results show a reduction in availability for a singleton in the presence of another singleton, it is not clear what caused this decrement. One potential explanation is that the reduced availability observed in the dual condition is a direct consequence of the context manipulation resulting in a cost at a perceptual level. That is, the presence of the additional singleton might have reduced the overall perceptual salience of both singletons in the dual compared to the single condition. Alternatively, the reduction might reflect the presence of response competition in the dual condition. That is, the presentation of two rather than one singleton in the dual condition could have induced a cost at a later stage of response selection. In this sense, the presence of an additional singleton might have affected the estimated availability of the singleton because of an increase in the number of response alternatives rather than because of a decrease in the perceptual salience of the individual singletons. Experiment 2 was performed to resolve this issue.

## Experiment 2

**Figure 5:**
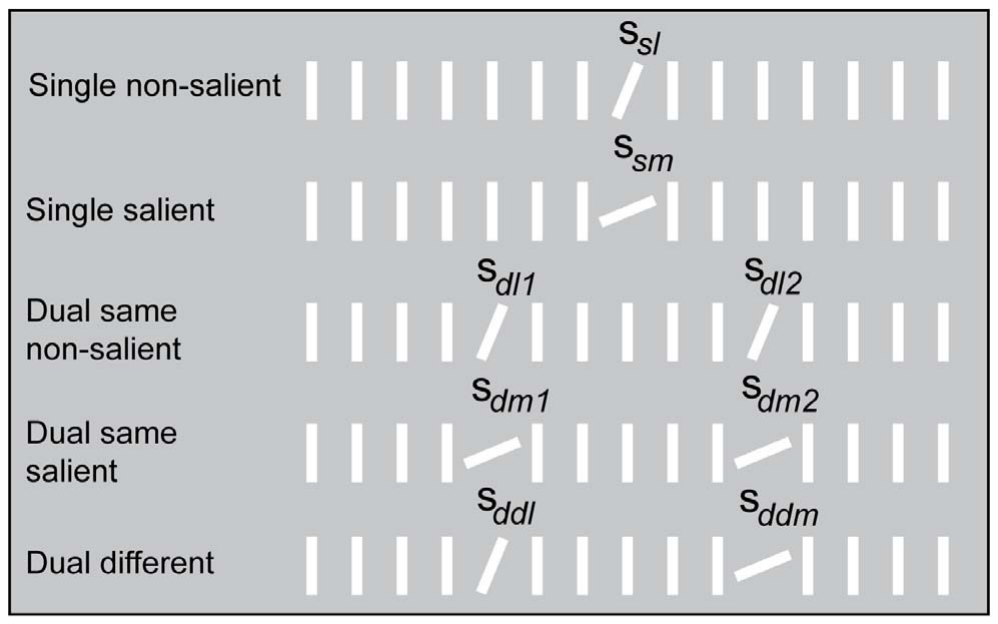
Stimulus overview of Experiment 2. The figure depicts a simplified overview of the different stimulus conditions. In the single conditions the singleton is either tilted 22.5° or 67.5° relative to the background lines. In the dual same conditions both singletons have an identical orientation contrast of either 22.5° or 67.5° whereas in the dual different condition the orientation contrast of the two singletons varies so that one is more salient (67.5°) than the other (22.5°) relative to the background lines.

The goal of Experiment 2 was to examine whether the difference in availability between the single and dual condition is due to a perceptual or a response related cost associated with the dual condition. To this end the stimulus salience of the singletons was varied across and within conditions. In one set of conditions the stimuli were identical to Experiment 1 (i.e., 22.5° relative to the background) whereas in another set of conditions the singletons in the single and dual conditions had a larger orientation contrast (i.e., 67.5° relative to the background). While in all four conditions the singletons were more salient than the background, the absolute difference in orientation contrast in the former set of conditions is smaller (therefore called single and dual non-salient conditions) than in the latter set of conditions (referred to as single and dual salient conditions). In a fifth condition, the dual different condition, the non-salient and salient singleton were simultaneously presented. For an overview of the different conditions in Experiment 2 see Figure 5.

If the difference in availability between the single and dual condition in Experiment 1 was due to perceptual differences, the availability of the singletons in the dual conditions should be affected by the salience manipulation. That is, the availability of a singleton should decrease when it is paired with another singleton and the size of this effect should depend on the salience of the other singleton. Note, that such a finding would not be in line with the notion of de Vries et al. (2011). According to this notion, a singleton's availability for selection is assumed to be a sole function of its local visibility, irrespective of the presence or salience of another (distant) singleton. Alternatively, if the difference was due to a difference in the number of response alternatives, the observed difference between single and dual conditions should be the same irrespective of the salience of the other singleton in the dual conditions.

### Method

#### Participants

Eleven participants (including the author AS) took part in the experiment in return for either money or course credit. Ages ranged from 19 to 28 years (mean: 23 years); eight of the participants were female. All participants reported normal or corrected-to-normal visual acuity and all apart from AS were naïve to the purpose of the study. The experimental session lasted for approximately 90 minutes.

#### Stimuli and Apparatus

The apparatus and visual stimuli were similar to the ones in Experiment 1 with the exception that rather than being identical, the orientation contrast of the singleton(s) relative to the orientation of the background lines (90° relative to the horizontal plane) was varied depending on the condition. In the single condition, the singleton was tilted either 22.5° (non-salient) or 67.5° (salient) clockwise relative to the background lines. The same was true for the dual same conditions with the difference that two identical singletons were presented in each condition. Finally, in the dual different condition, the orientation contrast of the singletons varied relative to each other, with one being non-salient (orientated 22.5° clockwise) and the other one salient (67.5° clockwise) relative to the background lines. Figure 5 presents an overview of the different presentation conditions in Experiment 2.

#### Design and Procedure

Design and procedure were identical to Experiment 1. Participants were instructed to make a speeded eye movement to the only orientation singleton in the single conditions and to either of the two singletons in the dual conditions. Participants performed 60 practice trials, which were not included in any analyses, and a total of 1200 experimental trials, 200 in each of the two single and two dual same conditions and 400 in the dual different condition. All trials were presented in randomized order, equally distributed across five blocks and separated by four small breaks.

#### Data Analysis

The criteria and procedure for data analysis were identical to the one in Experiment 1. However, rather than being distributed over two conditions, trials were now distributed across five different conditions. In order to consider the factor salience, analyses were adjusted accordingly.

### Results

A total of 14.8% of all trials was excluded from further analyses (3.9% due to anticipation errors, 0.4% due to attention-lapse errors, 7.3% due to saccade destination errors and on 3.2% the amplitude remained below 2.3°). No erroneous blinks were recorded in the time interval from display onset until fixation of a singleton and none of the participant's datasets reached the critical removal criterion of 30%.

In order to examine differences in timing of selection between the different presentation conditions as well as between the two different levels of salience, we performed a univariate ANOVA with Condition (single, dual same and dual different) and Salience of the selected element (salient and non-salient) as within-subject factors on the averaged individual saccade latencies. The results only showed a marginally significant effect of Salience [*F*(1, 60) = 4.688, *η* ^2^ = .002, *p* = .05], attributable to participants being somewhat faster at selecting the more salient [*m* = 193.5 ms] than the less salient singleton [*m* = 211.9 ms]. However, individual comparisons between Salience within every presentation condition revealed that the main effect of Salience vanished when separately comparing the latencies of the two single [*F*(1, 20) = 3.124, *η* ^2^ = .002, *p* = .161], the two dual same [*F*(1, 20) = 0.936, *η* ^2^ = .001, *p* = .345] and the two singletons in the dual different conditions [*F*(1, 20) = 1.271, *η* ^2^ = .002, *p* = .273].

**Figure 6:**
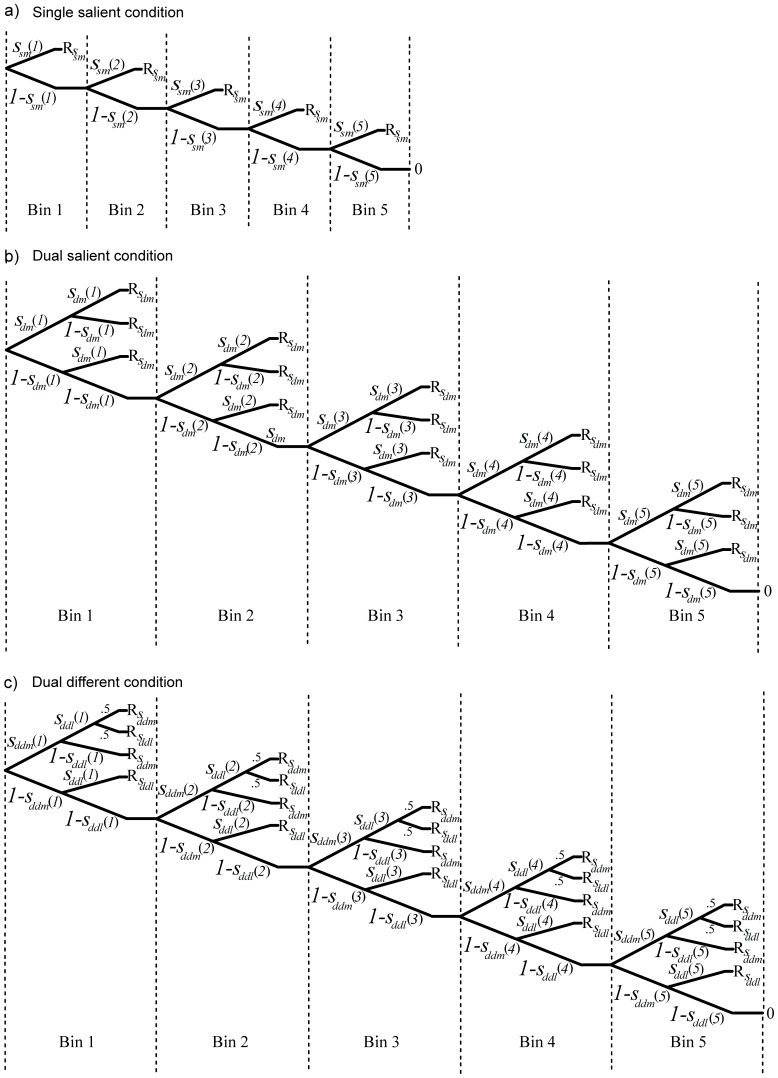
Tree diagrams in Experiment 2. The tree diagrams correspond to a) the single, b) the dual same, and c) the dual different condition and depict the probabilities that the singleton is available for selection (italics) for each bin (in brackets), leading to a given response outcome (R) for each individual trial. The response outcome is determined through the path leading to it, which equals the product of probabilities on that path. Note that, for reasons of simplicity, the diagrams in a) and b) correspond to the salient singleton only. 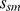 denotes the probability that the singleton is available in the single most condition, 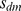 denotes the probability that the singleton is available in the dual most condition, and 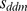 and 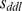 denote the probability that the singleton is available in the dual different condition, for the more and less salient singleton, respectively.

Analogously to Experiment 1, we calculated the availability values of each singleton separately per condition with the assumption that the availability of the singletons within a given dual same condition was identical. The tree diagram on which the calculation is based is shown in Figure 6.

**Figure 7:**
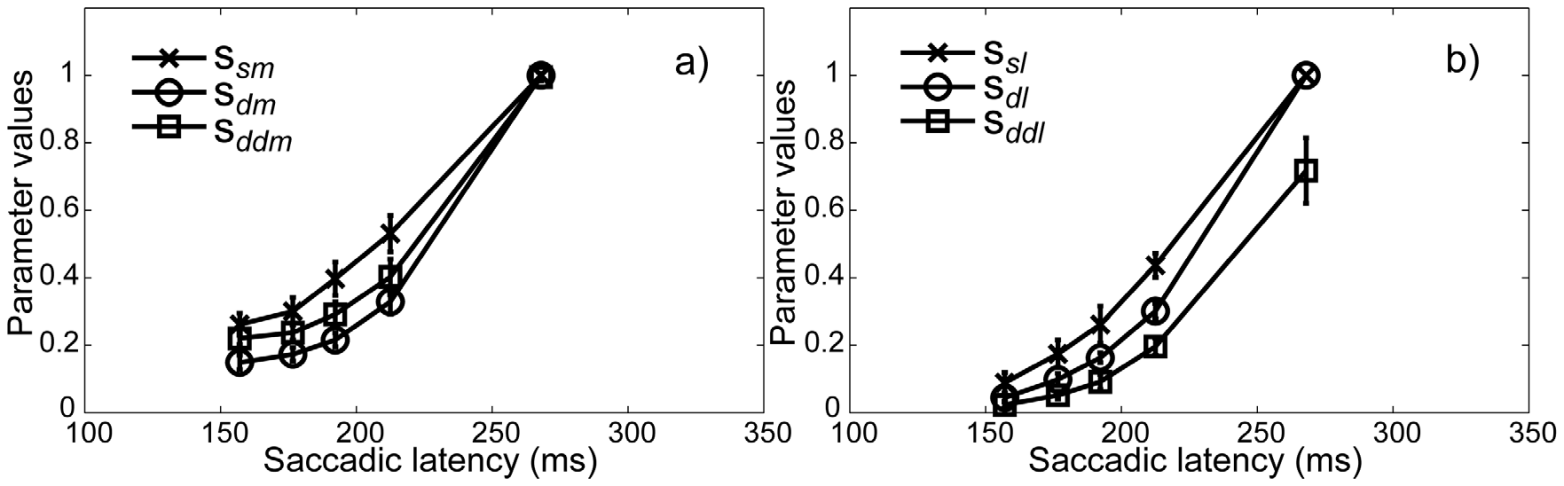
Parameter values in Experiment 2. The parameter values represent the probability that a singleton is available for selection separately per condition for each Bin *i*, averaged over participants. The values in a) correspond to the parameter estimates obtained for the salient singleton for each of the three conditions, whereas b) depicts the parameter estimates for the non-salient singleton for each of the three conditions. Error bars represent 95% confidence intervals. Note that based on the assumption that the availability of both singletons within each dual same condition is identical, Bin 5 in these conditions adds up to 1 by definition.

A 3 (Condition) * 2 (Salience) * 5 (Saccadic Latency Bin) repeated measures ANOVA was performed on the availability values corresponding to each singleton. The results revealed main effects of Condition [*F*(2, 20) = 95.820, *η* ^2^ = .027, *p*<.001], Salience [*F*(1, 10) = 97.275, *η* ^2^ = .037, *p*<.001] and Saccadic Latency Bin [*F*(4, 40) = 6194.991, *η* ^2^ = .883, *p*<.001] as well as significant interactions between Condition and Salience [*F*(2, 20) = 52.963, *η* ^2^ = .011, *p*<.001], Condition and Saccadic Latency Bin [*F*(8, 80) = 14.252, *η* ^2^ = .010, *p*<.001], Salience and Saccadic Latency Bin [*F*(4, 40) = 2.711, *η* ^2^ = .001 *p*<.05], and Condition, Salience and Saccadic Latency Bin [*F*(8, 80) = 7.126, *η* ^2^ = .004 *p*<.001]. Post-hoc Bonferroni corrected comparisons revealed that, overall, the probability that the salient singleton was available was higher [*m* = .43, *std error* = .006] than the probability that the non-salient singleton was available [*m* = .31, *std error* = .007, *p*<.001]. Separate analyses within each condition showed that this was the case for each of the three conditions [*F*(1, 10) = 34.575, *η* ^2^ = .030, *p*<.001 for the single, *F*(1, 10) = 33.437, *η* ^2^ = .006, *p*<.001 for the dual same and *F*(1, 10) = 140.974, *η* ^2^ = .126, *p*<.001 for the dual different condition]. Furthermore, the probability that the singleton in the single conditions was available was overall higher [*m* = .45, *std error* = .008] than in the dual same conditions [*m* = .35, *std error* = .004, p<.001] and in the dual different condition [*m* = .32, *std error* = .003, *p*<.001]. While overall the probability of availability in the dual same conditions was also significantly higher than in the dual different condition [*p*<.001], the interaction between Condition and Salience showed that this difference was purely due to a significantly lower probability for the non-salient singleton in the dual different condition [*m* = .22] compared to the non-salient singletons in the single [*m* = .39] and dual same condition [*m* = .32]. In fact, while lower than in the single condition [*m* = .5, *std error* = .013], the probability that the salient singleton in the dual different condition was available was higher [*m* = .43, *std error* = .008] than in the dual same condition [*m* = .37, *std error* = .008]. Figure 7 depicts a graph of the results separately for the salient and non-salient singleton per condition.

Finally, we performed a cross-experimental comparison in order to investigate whether the effect of Condition (single versus dual same) is equivalent for both Experiment 1 and 2. The results of the univariate ANOVA showed a main effect of Condition [*F*(1, 58) = 211.668, *η* ^2^ = .013, *p*<.001] and a marginally significant interaction between Condition and Salience [*F*(1, 58) = 4.010, *η* ^2^ = .000, *p* = .05]. The effect of Experiment did not reach significance [*F*(1, 58) = 0.081, *η* ^2^ = .000, *p* = .776], suggesting that the results did not differ between the two experiments.

### Discussion

The results of Experiment 2 replicated the finding of Experiment 1: overall, the availability of the singleton in any of the single conditions was higher than in the corresponding dual same condition, showing that the probability that a singleton is available is reduced in the presence of an additional identical singleton relative to single presentation. Crucially, the availability of a given singleton in the dual conditions was differentially affected depending on the absolute orientation contrast of the other singleton relative to the background lines. This finding supports the idea that a singleton's availability for oculomotor selection depends on the local feature contrast of the simultaneously presented other singleton, thus the relative salience between elements. It is important to note that in Experiment 1 and all conditions in Experiment 2 apart from the dual different condition, the parameter estimates corresponding to the last bin are 1 by definition. Since the distribution in the dual different condition in Experiment 2 consists of the combined responses to the salient and non-salient singleton, the parameter estimate corresponding to the non-salient singleton did not reach one in the dual different conditions. This result illustrates that the availability of the non-salient singleton for oculomotor selection was substantially reduced by the presence of the (distant) salient singleton: the salient singleton was prioritized over the non-salient singleton, even in the bin corresponding to the long-latency saccades (i.e., the fifth bin). More importantly, this finding is not reconcilable with the idea that the cost in availability of a singleton in the dual compared to the single condition is the result of a response-related cost. If this would have been the case, the observed costs in availability should have been the same when comparing the different dual conditions to the single conditions. Thus, it seems that the presence of an additional singleton negatively affects the probability that this singleton becomes perceptually available for selection. Moreover, the effect of an additional singleton was demonstrated to be critically dependent of its salience. A more salient singleton has a larger effect than a less salient singleton.

## Conclusions

The goal of the current study was to investigate time-dependent effects of the number of targets presented and stimulus salience on oculomotor selection behavior. To this end, observers had to make a speeded eye movement to a target orientation singleton embedded in a homogeneous background of vertically oriented lines. In Experiment 1, either one or two physically identical targets were presented whereas in Experiment 2 an additional salience manipulation was performed in which the singleton(s) had a small or large orientation contrast relative to the background lines. These differently salient singletons were either presented individually or together.

The results of both experiments showed two important findings that help clarify the relationship between salience-processing in terms of local feature contrast, its relationship with other elements presented elsewhere in the display (the relative salience between elements) and its effects on oculomotor selection behavior. These findings are:

1. the availability of a singleton in the presence of an identical singleton relative to single presentation is reduced.
2. Moreover, the size of the reduction was directly related to the local feature contrast of the simultaneously presented singleton, i.e., a singleton paired with a salient singleton led to a larger reduction than a singleton paired with a less salient singleton. Furthermore, while in the dual same conditions the relative salience between the singletons was identical, the dual different condition provided insight into the effect of the relative salience between elements. The results showed that in addition to the local feature contrast of the individual elements, the relative salience between elements has a large effect on the selection probability of an individual singleton.

Together, these results provide evidence against an absolute processing speed account. According to this account, an individual item has a particular speed at which it is processed and differences in the absolute processing speed of individual elements determine selection preferences. That is, the probability that the salient singleton is available for selection should have been the same irrespective of whether it is presented alone, together with an identical singleton or with a less salient singleton. The same applies to the non-salient singleton. However, the results of the current study showed that rather than a particular singleton's inherent visibility, the probability that a given singleton is available for selection is mainly determined by the broader context in which the singleton is embedded. In fact, we do not argue that the visibility of a singleton is not important in determining its availability for selection. The results clearly show that overall the singleton with a larger orientation contrast relative to the background is more likely to be available than the singleton with the smaller orientation contrast. This is in line with the finding that a larger local feature contrast generates more activity in the salience map than a smaller feature contrast [37]–[39]; [41]. However, rather than the local feature contrast, which represents the immediate surroundings of a singleton, the major determinant of whether a singleton is available or not is the relative salience between elements. The fact that a singleton affects the availability of an equally or less salient singleton, even when presented multiple degrees apart, highlights the importance of the more distant display context of the singleton in determining its availability.

In sum, we showed that the presence of an additional singleton reduces the availability of both singletons for selection. The finding that relative stimulus salience modulated the effect of number of targets suggests that the presence of multiple targets induces a cost at an early perceptual rather than at a later response decision stage. This highlights that not only the immediate context of an item as measured by local feature contrast but the more distant environment which has a major effect on how these items are perceived. While two identical elements (equally salient) reduce the overall availability of each element, the availability of a given element is compromised even more if the salience of the other element is higher.
